# Long Noncoding RNAs Regulate Hyperammonemia-Induced Neuronal Damage in Hepatic Encephalopathy

**DOI:** 10.1155/2022/7628522

**Published:** 2022-02-21

**Authors:** So Yeong Cheon, Danbi Jo, Young-Kook Kim, Juhyun Song

**Affiliations:** ^1^Department of Biotechnology, College of Biomedical & Health Science, Konkuk University, Chungju, Republic of Korea; ^2^Department of Anatomy, Chonnam National University Medical School, Hwasun, Jeollanam-do, Republic of Korea; ^3^BioMedical Sciences Graduate Program (BMSGP), Chonnam National University, Hwasun, Jeollanam-do, Republic of Korea; ^4^Department of Biochemistry, Chonnam National University Medical School, Hwasun, Jeollanam-do, Republic of Korea

## Abstract

**Background:**

Hyperammonemia can result in various neuropathologies, including sleep disturbance, memory loss, and motor dysfunction in hepatic encephalopathy. Long noncoding RNA (lncRNA) as a group of noncoding RNA longer than 200 nucleotides is emerging as a promising therapeutic target to treat diverse diseases. Although lncRNAs have been linked to the pathogenesis of various diseases, their function in hepatic encephalopathy has not yet been elucidated. *Research Design and Methods*. To identify the roles of lncRNAs in hepatic encephalopathy brain, we used a bile duct ligation (BDL) mouse model and examined the alteration of neuronal cell death markers and neuronal structure-related proteins in BDL mouse cortex tissue. Furthermore, analysis of the transcriptome of BDL mouse brain cortex tissues revealed several lncRNAs critical to the apoptosis and neuronal structural changes associated with hepatic encephalopathy.

**Results:**

We confirmed the roles of the lncRNAs, ZFAS1, and GAS5 as strong candidate lncRNAs to regulate neuropathologies in hepatic encephalopathy. Our data revealed the roles of lncRNAs, ZFAS1, and GAS5, on neuronal cell death and neural structure in hyperammonemia in *in vivo* and *in vitro* conditions.

**Conclusion:**

Thus, we suggest that the modulation of these lncRNAs may be beneficial for the treatment of hepatic encephalopathy.

## 1. Introduction

Hepatic encephalopathy is considered as a consequence of liver failure, including in cases of severe liver cirrhosis [1]. Hepatic encephalopathy reduces the quality of life and can result in sustained economic losses for patients who require repeated hospitalization [2]. Hepatic encephalopathy is a multifactorial neurological syndrome accompanied by cognitive impairment, brain edema, coma, somnolence, and motor dysfunction [1, 3]. Although the factors affecting hepatic encephalopathy are varied and include genetic factors, environment, and dietary habits, the major factor in its pathogenesis is an increase in ammonia accumulation and abnormal neurotransmitter secretion in the brain [4, 5]. Also, ammonia levels in the blood and cerebrospinal fluids were elevated in the patients with hepatic encephalopathy [6].

When excess ammonia passes the blood-brain barrier, it can promote astrocyte swelling and neuronal cell dysfunction [7]. Thus, hyperammonemia in the central nervous system (CNS) is a key cause in the neuropathogenesis of hepatic encephalopathy [8]. A high level of ammonia triggers neuronal cell death, induces oxidative stress [9], and inhibits axonal outgrowth [10]. In addition, hyperammonemia causes brain atrophy, demyelination, and reduced production of neurotransmitters, such as glutamate [11], and excessive free radical generation has been linked to a diverse range of neuropathologies [12]. A previous study has shown that a high level of ammonia results in hippocampal neuronal cell death mediated by B-cell lymphoma 2- (BCL2-) associated agonist of cell death (BAD) dephosphorylation [13], ultimately leading to the progression of several neurodegenerative diseases, including Alzheimer's diseases [14].

Long noncoding RNAs (lncRNAs) are a group of noncoding RNAs of more than 200 nucleotides in length that do not harbor any protein-coding sequences. These transcripts operate by modulating the transcription of protein-coding genes in the nucleus or inhibiting the binding of microRNAs in the cytoplasm. LncRNAs have been linked to the pathogenesis of several diseases, but their involvement in hepatic encephalopathy has not yet been elucidated.

In this study, we investigated whether hyperammonemia contributes to apoptosis signaling in neurons using both *in vitro* and *in vivo* experiments. In addition, we identified several candidate lncRNAs related to ammonium-induced neuronal cell death and confirmed the role of some of these lncRNAs in neuronal cell death and structural change. Our data support the inclusion of these lncRNAs in the development of potential therapeutics against hepatic encephalopathy.

## 2. Materials and Methods

### 2.1. Animals Subjected to BDL

Male C57BL/6 mice, aged 12-14 weeks, were purchased from Orient Bio (Seongnam, Gyeonggi-do, South Korea) and used in this study. All mice were housed under controlled conditions with a 12 h light/dark cycle and constant temperature. The animals were provided free access to food and water. Mice were randomly assigned to the control or bile duct ligation (BDL) group and then anesthetized using 5% isoflurane in mixed gas and maintained with 2% isoflurane during surgery. Mice were placed on a temperature control blanket, and their abdominal fur was removed using a shaver. The skin was sterilized with 70% ethanol, and the abdomen was dissected using surgical scissors. Once opened, the bile duct was ligated using a 5-0 silk suture. After ligation, the peritoneum and abdominal skin were closed with 5-0 silk sutures and sterilized with 70% ethanol. Mice were returned to their home cages and then sacrificed 14 days after surgery. Body weight was measured before and after BDL. Mouse brains were collected following cardiac perfusion with sterile saline, and the cerebral cortex was separated and stored at -70°C until use. The experiments were performed following the recommendations of the 96 guidance for animal experiments established by the Animal Ethics Committee at Chonnam National University (CNU). The Animal Ethics Committee approved the protocol at CNU. The study was carried out in compliance with the ARRIVE guidelines.

### 2.2. Cell Culture Conditions

The human neuroblastoma cell line (CRL-2266, ATCC), SH-SY5Y, was cultured in Minimum Essential Medium (MEM) supplemented with 10% fetal bovine serum (FBS), 100 U/ml penicillin-streptomycin, and 1 mM sodium pyruvate. A total of 4.0 × 10^4^ SH-SY5Y cells/cm^2^ were seeded onto cell culture plates and incubated at 37°C with 5% CO_2_. After 1 day of seeding, the cell culture medium was replaced with a differentiating medium containing 2% FBS, 100 U/ml penicillin-streptomycin, and 20 *μ*M all-trans-retinoic acid for 2 days. In the lncRNA knockdown experiments, SH-SY5Y cells were transfected with 30 nM of each siRNA, and after 1 day, the cells were treated with 30 mM NH_4_Cl (Sigma-Aldrich, A9434) for 24 h to induce hyperammonemia. This culture schedule is described in Supplementary Figure S1A.

### 2.3. RNA Sequencing and Analysis

RNA sequencing and analysis were performed as previously described [15]. Briefly, total RNA was prepared from the cerebral cortex using TRIzol (Thermo Fisher Scientific), and ribosomal RNA was removed using a Ribo-Zero rRNA depletion kit. The sequencing library was prepared using TruSeq Stranded Total RNA preparation kit (Illumina) and applied to a NovaSeq 6000 System (Illumina) for paired-end sequencing with 100 sequencing cycles.

The sequencing reads were quality-checked, and low-quality reads were trimmed using Trimmomatic [16]. We then used two different approaches to calculate the transcript levels and combined these results to select a reliable subset of transcripts for functional analysis as previously described [15]. For the first approach, the filtered reads were aligned against the mouse genome (mm10) using STAR aligner, and the expression levels of the coding and noncoding RNAs were calculated using Cuffnorm [17], which is based on the GENCODE annotation [18]. The transcripts with average fragments per kilobase of transcript per million mapped reads (FPKM) lower than 1 or those with FPKM = 0 in any sample were removed from further analysis. The expression change of each gene was calculated, and the *P* value was determined using Student's *t*-test. For the second approach, transcript levels were quantified using the Salmon algorithm [19], and differential expression was calculated using the edgeR package [20]. Finally, the transcripts belonging to the top 10% (based on *P* value) of the transcripts identified in each approach were selected for downstream analysis.

We performed unsupervised hierarchical clustering using Cluster 3.0 and FPKM values from the Cuffnorm analysis and then visualized the clusters using Java Treeview. [21, 22] The functional analysis for both the upregulated and downregulated transcripts was performed using hallmark and GO analyses in the Molecular Signatures Database (MSigDB) [23].

The differentially expressed lncRNAs were selected by comparing the significantly upregulated or downregulated genes from the cortex of the BDL and normal mice. First, we removed any protein-encoding transcripts, small RNA genes, and immunoglobulin-associated genes. This left the lncRNA transcripts, which were further narrowed to only those lncRNAs that appeared in the top 10% (based on *P* value) of transcripts in both the STAR-Cuffnorm and Salmon-edgeR evaluations. This process eventually identified eight high-value lncRNA transcripts.

### 2.4. Quantitative Reverse Transcription-Polymerase Chain Reaction (RT-PCR)

Quantitative RT-PCR was used to confirm the knockdown efficiency of the siRNAs in the SH-SY5Y cells. Total RNA was extracted from siRNA-transfected cells using TRIzol reagent (Ambion, Austin, TX, USA) according to the manufacturer's instructions. cDNA was synthesized using RevertAid reverse transcriptase (Thermo Fisher Scientific, Waltham, MA, USA) and random hexamers, and RNA expression was measured using Power SYBR Green PCR Master Mix and a Step One Plus Real-Time PCR System (Applied Biosystems, Foster City, CA, USA). Each sample was normalized against glyceraldehyde 3-phosphate dehydrogenase, and the primer sequences used in these assays are presented in Supplementary Figure S1B.

### 2.5. siRNA Design and Transfection

The siRNAs against the lncRNAs were designed using the siDESIGN Center on the horizon discovery platform (https://horizondiscovery.com/en/products/tools/siDESIGN-Center) and i-Score Designer (https://www.med.nagoya-u.ac.jp/neurogenetics/i_Score/i_score.html). The AccuTarget negative control siRNAs (Bioneer) were used as a negative control, and 4.0 × 10^4^ cells/cm^2^ were transfected with a final concentration of 30 nM siRNA per well using Lipofectamine 3000 (Invitrogen, Carlsbad, CA, USA) according to the manufacturer's instructions and incubated for 48 h prior to analysis. The siRNA sequences are presented in Supplementary Figure S1C.

### 2.6. Western Blotting

SH-SY5Y cells were lysed in radioimmunoprecipitation assay buffer (TransLab, Daejeon, South Korea). Protein extracts were quantified using a BCA assay kit (Thermo Fisher Scientific, Waltham, MA, USA) according to the manufacturer's instructions. Protein lysate (25 *μ*g) was electrophoresed on 10-15% sodium dodecyl sulfate (SDS)-polyacrylamide gels and transferred to polyvinylidene difluoride (PVDF) membranes (Merck Millipore, Burlington, MA, USA) activated by methanol. The membranes were blocked with 5% skim milk or 5% BSA in 1X TBS-T (10X TBS buffer and 0.1% Tween-20) for 1 h at room temperature and then incubated with the appropriate primary antibodies (1 : 1000 dilution) at 4°C overnight. After incubation, the membranes were washed with 1X TBS-T buffer three times for 10 min each and then placed in 1X TBS-T buffer containing secondary antibodies (1 : 2000 dilution) for 2 h at room temperature. These membranes were then washed with 1X TBS-T buffer three more times and visualized with ECL solution (Thermo Fisher Scientific) and Fusion Solo software (Vilber, Marne-la-Vallée, France). The protein expression levels were quantified using ImageJ software and normalized against those of *β*-actin.

For the western blot analyses performed using mouse cortical tissues, protein lysates were prepared using tissue protein extraction reagent and protease and phosphatase inhibitors (Halt™ Protease and Phosphatase Inhibitor Cocktail, Thermo Fisher Scientific) and then homogenized with a pestle. Tissue lysates were allowed to react on ice for 5 min and centrifuged at 13,000 rpm at 4°C for 30 min. After centrifugation, the supernatants were collected, and protein concentration was evaluated using a Pierce® BCA protein assay kit (Thermo Fisher Scientific). Proteins were separated on 8–15% SDS-PAGE and then transferred to PVDF membranes (Merck Millipore). The membranes were incubated with primary antibodies (Supplementary Figure S1D), washed, and then incubated with the HRP-conjugated secondary antibodies from Santa Cruz Biotechnology. Protein levels were visualized using a chemiluminescence system (Immobilon® Western Chemiluminescent HRP substrate; EMD Millipore Corporation, Burlington, MA, USA, or West-Q pico Dura ECL solution; GenDEPOT, Barker, TX, USA), and the ImageQuant LAS 4000 (GE Healthcare, Pittsburgh, PA, USA) was used to determine protein density, which was compared using the ImageJ program.

### 2.7. Ammonia Measurements

To measure the levels of ammonia in mouse cerebral cortex and SH-SY5Y neuronal cells, cerebral cortex was isolated with or without BDL, and SH-SY5Y supernatants and cell lysates were collected following transfection with the relevant siRNAs with or without NH_4_Cl treatment. The level of ammonia was determined using PocketChem™ BA (Arkay Inc., Kyoto, Japan) according to the manufacturer's instructions.

### 2.8. Cell Viability Assay

NH_4_Cl-induced cytotoxicity was measured using a WST assay. SH-SY5Y cells were seeded onto 96-well plates at a density of 5.0 × 10^4^ cells/well. Cell viability was then evaluated 24 h after NH_4_Cl treatment by adding 10 *μ*L/well of the Ez-CyTox reagent and incubating for 1 h at 37°C. Fluorescence intensity was measured at a wavelength of 450 nm using an Epoch microplate reader (BioTek, Winooski, VT, USA).

### 2.9. Measurement of Cell Death

Cell death in the brain with or without BDL was measured by using cell death detection ELISA kit from Roche (Basel, Switzerland). It detects cytoplasmic histone-associated DNA fragments. Briefly, isolated proteins from the cerebral cortex were prepared, incubated in anti-histone antibody-coated microplate module, and reacted with anti-DNA-POD in each well following the manufacturer's protocol. After all procedures, the microplate was measured at a wavelength of 405 nm using a microplate reader (Molecular Devices, Sunnyvale, CA, USA).

### 2.10. Lactate Dehydrogenase (LDH) Assay

Lysates from cerebral cortex tissues after BDL were added to a 96-well plate to measure lactate dehydrogenase **(**LDH) levels using an LDH assay kit from Invitrogen. The reaction mixture in the assay kit was added to each well. After 30 min of incubation, the stop solution was added to each well, and absorbance (at 490/680 nm) was measured by using a microplate reader (Molecular Devices).

### 2.11. Enzyme-Linked Immunosorbent Assay (ELISA)

The levels of interleukin (IL)-6 and tumor necrosis factor (TNF)-*α* in the cerebral cortex were measured by ELISA assay (R&D systems, Minneapolis, MN, USA). Cortical tissues were prepared and added to each well, and assay diluents were then added according to the manufacturer's instructions. The samples were incubated at 4°C overnight. In the next day, samples were washed with wash buffer and incubated with mouse IL-6 or TNF-*α* conjugates. The absorbance at 450 nm was measured by using a microplate reader (Molecular Devices).

### 2.12. Neurite Outgrowth and Complexity

SH-SY5Y cells (4.0 × 10^4^ cells/cm^2^) were stained with the neurite outgrowth staining kit (Life Technologies, Carlsbad, CA, USA), according to the manufacturer's instructions. The cells were incubated in 1X cell membrane stain mixture in 4% paraformaldehyde for 20 min at 37°C and then placed in 1X background suppression dye in phosphate-buffered saline (PBS). Neurite outgrowth (*λ*ex 555 nm/*λ*em 565 nm) was visualized using an Eclipse Ts2 fluorescent microscope (Nikon, Tokyo, Japan). Fluorescence intensities were measured using ImageJ software. Neuronal complexities (neurite length, number of secondary branches, and number of neurites from the soma) were analyzed in a blinded manner and evaluated using ImageJ software. Neurite length data were normalized against those of the control group.

### 2.13. 3,3′-Diaminobenzidine Staining

The mouse brain tissues used in the immunohistochemistry experiments were first perfused and then fixed in 4% formaldehyde. The sections were then permeabilized using 0.3% Triton X-100 for 30 min at room temperature and incubated in methyl alcohol for 10 min at –20°C. Endogenous peroxidase activity was blocked by treating the samples with hydrogen peroxide (0.3%). These samples were then washed in PBS and incubated in blocking solution for 30 min at room temperature and immunolabeled with primary antibodies against microtubule-associated protein 2 (MAP2) overnight at 4°C. The sections were then washed with PBS and incubated with HRP-conjugated secondary antibodies diluted in PBS for 1 h at room temperature. Immunoreactive cells were visualized as a dark brown color using 3,3′-diaminobenzidine staining (Vector Laboratories, Inc., Burlingame, CA, USA). MAP2-positive signals were observed using a microscope coupled with a digital camera (Olympus, Tokyo, Japan).

### 2.14. Statistical Analysis

The data are presented as the mean ± standard error of the mean (SEM). The statistical comparisons between the *in vivo* control and BDL groups were measured using an unpaired *t*-test in GraphPad Prism Software v7.0 (GraphPad Software, San Diego, CA, USA). *P* < 0.05 was considered statistically significant.

## 3. Results

### 3.1. The Murine Bile Duct Ligation (BDL) Model Exhibits Hyperammonemia and Pathological Condition in the Cerebral Cortex

**Figure 1:**
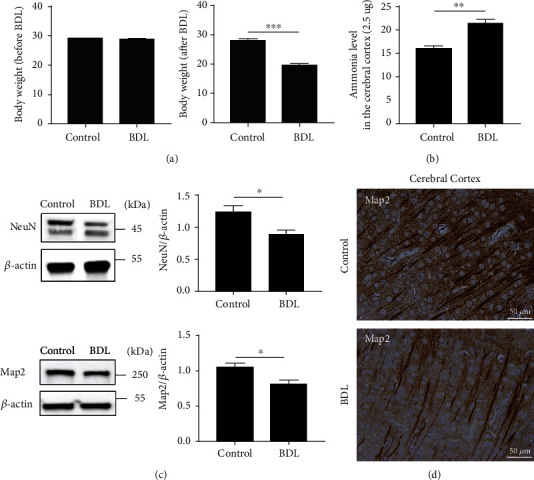
BDL mice display hyperammonemia and pathological condition in the cerebral cortex. (a and b) Body weight before and after the surgery and the ammonia level in the cerebral cortex with or without BDL. (c) Western blot showing the protein levels of neuronal marker and structure markers, NeuN and Map2 in the murine cerebral cortex at two weeks after BDL surgery. The level of each protein was normalized against that of *β*-actin. (d) Immunohistochemistry for the neuronal structure marker, Map2, in murine cerebral cortex tissues at two weeks after BDL surgery. In (a)–(d), the error bars represent S.E.M. from three or four mice. ^∗^*p* < 0.05, ^∗∗^*p* < 0.01, and ^∗∗∗^*p* < 0.001 (unpaired *t*-tests).

To examine a detailed molecular change in the brain cortex during the progression of hepatic encephalopathy, we established a mice BDL model. Two weeks later of BDL, the bodyweight of mice was significantly decreased compared to normal mice (Figure 1(a)). It has been reported that hepatic encephalopathy is characterized by hyperammonemia and neuronal dysfunction [6, 24]. Therefore, we checked the level of ammonia in the cerebral cortex and confirmed the increase of ammonia level after BDL (Figure 1(b)). To confirm whether BDL affects the expression of a neuronal marker protein in cerebral cortex tissue, we first evaluated the expression of NeuN, a neuronal marker to assess a pathological condition of neurons. When the cerebral cortex tissues in BDL mice were examined two weeks after injury, we noted that the BDL mice cortex tissue showed decreased levels of NeuN compared to the control suggesting the pathological state of neurons in BDL mice (Figure 1(c)). Map2, a member of the microtubule-associated protein family and a cytoskeletal element, is abundantly expressed in neuronal cells and plays an essential role in the stabilization of the microtubule, neuronal outgrowth, and neuronal cell death [25]. The level of Map2 was decreased in the cortex from BDL mice (Figure 1(c)). Immunohistochemistry data also exhibited fewer Map2-positive cells in BDL mice cortex tissue compared to the control (Figures 1(d) and S2). Therefore, our results indicate the cerebral cortex tissue of BDL mice as a proper model of hepatic encephalopathy and show hyperammonemia and pathological condition of the cerebral cortex in BDL model.

### 3.2. Transcriptomic Analysis of a Mouse Model of Hepatic Encephalopathy

**Figure 2:**
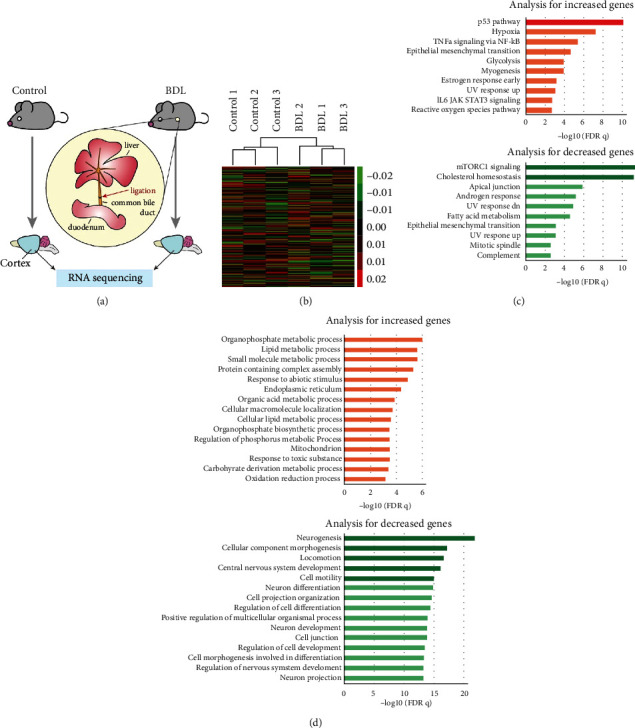
Transcriptome profiling of the cortex tissues from a BDL mouse model. (a) The experimental procedure used to perform the RNA sequencing experiments on the mouse cortex tissues (see Materials and Methods for more information). (b) Hierarchical clustering based on gene expression data produced using RNA sequencing. (c) Hallmark analysis using the differentially expressed genes in MSigDB. The top 10 hallmarks were selected based on their FDR *q* value. The terms with negative log values of more than 10 are indicated by a darker bar. (d) Gene ontology (GO) analysis using the differentially expressed genes in MSigDB. The top 15 GO terms were identified using their FDR *q* value. The terms with negative log values of more than 15 are indicated by a darker bar.

Next, we performed RNA sequencing analysis of the brain cortex tissues collected from normal and BDL mice to confirm whether the changes in the brain tissues identified above were also observed at the transcriptomic level (Figure 2(a)). RNA sequencing analysis allowed us to evaluate the transcription of both protein-coding and noncoding RNAs. The hierarchical clustering analysis of the differentially expressed transcripts revealed that the normal and BDL mouse tissue samples produced distinct clusters (Figure 2(b)).

We then identified the altered biological pathways using the hallmark analysis available at the MSigDB [23]. The upregulated genes were implicated in the p53 and hypoxia pathways, while the downregulated genes were enriched for those pathways associated with mTOR complex 1 (mTORC1) signaling and cholesterol homeostasis (Figure 2(c)).

We also performed gene ontology (GO) analysis to identify enriched terms in each of the upregulated and downregulated gene groups. The significantly enriched terms were primarily associated with the downregulated genes and included neurogenesis, cellular component morphogenesis, locomotion, and central nervous system development (Figure 2(d)). This result is consistent with our experimental data showing that the expression of NeuN and Map2 is decreased in the BDL model (Figures 1(c) and 1(d)).

### 3.3. The BDL Mice Model Faithfully Reflects the Alteration of Biological Pathways Identified from Transcriptomic Analysis

**Figure 3:**
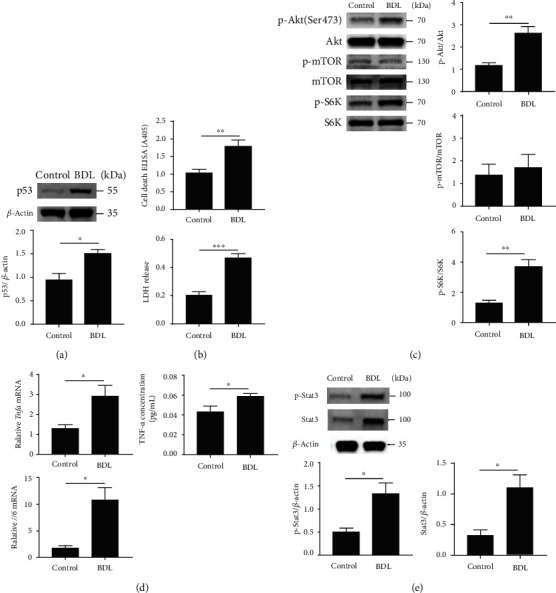
The BDL mice model shows alteration of biological pathways identified from transcriptomic analysis. (a) Expression change of the p53 protein level in the cerebral cortex of BDL mice at two weeks after BDL surgery. The level of each protein was normalized against that of *β*-actin. (b) Measurement of cell death and LDH release in control and BDL mice groups. (c) The components of the ammonia-related signaling pathways (Akt, mTOR, and S6K) were measured by western blot analysis. Phosphorylated forms of Akt, mTOR, and S6K were normalized against their total protein content. (d) The mRNA and protein levels of IL-6 and TNF-*α* after BDL surgery. (e) The protein levels of p-Stat3 and Stat3 in the cerebral cortex after BDL surgery. In (a)-(e), the error bars represent S.E.M. from three or four mice. ^∗^*p* < 0.05 and ^∗∗^*p* < 0.01 (unpaired *t*-tests).

Consistent with the results of transcriptomic analysis (Figure 2(c)), the cerebral cortex tissue in the BDL model showed increased p53 levels compared to the control (Figure 3(a)). Because p53 is a well-established inducer of apoptosis in various cell types, the increase in the levels of p53 protein suggests that neuronal cell death may be increased in BDL mice. As expected, the brain cortex tissues from BDL mice showed increased cell death and LDH release, compared to the control group (Figure 3(b)).

It has been reported that the high level of ammonium induces p53 signaling and promotes the Akt-dependent activation of mTORC1 in brain tissue [26, 27]. However, there is no fully elucidated exact evidence that ammonia-induced p53, Akt, and mTORC1 signalings are involved in neuronal cell death [28]. To confirm this hypothesis, we measured the levels of Akt and mTORC1 components in the cerebral cortex tissue of a murine BDL model. Western blot analysis was performed at two weeks following BDL surgery and showed an increased level of p-Akt (Ser 473) in the cerebral cortex tissue compared to the control group. Although there were no differences in the expression levels of the mTOR complex between these two groups, we observed an increase in the levels of the mTORC1 substrate, p70S6 kinase (S6K) in the cortex of the BDL mice model, compared to the control (Figure 3(c)).

The transcriptomic analysis also suggested an increase of inflammation in the brain cortex from BDL mice based on the increase of TNF-*α* and IL-6-associated pathways. The mRNA level of IL-6 was efficiently elevated after BDL surgery. The protein level of IL-6 after BDL surgery showed an increased tendency compared to the control, albeit less significant (Figures 3(d) and S3). We also detected the increased level of TNF-*α* mRNA and protein in the cerebral cortex compared to the control (Figure 3(d)).

Finally, signal transducer and activator of transcription 3 (Stat3) level were measured in the cerebral cortex following BDL surgery. The protein levels of pStat3 and Stat3 were significantly increased in the BDL mice compared to the control group (Figure 3(e)). Thus, our data successfully confirmed the results of the transcriptomic analysis of the BDL model.

### 3.4. Selection of lncRNAs Involved in the Pathogenesis of Hepatic Encephalopathy

**Figure 4:**
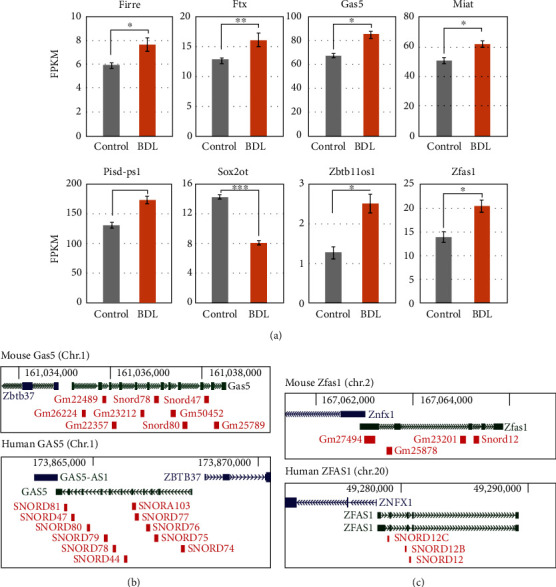
Selection of differentially expressed long noncoding RNAs in the brain cortex of BDL mice. (a) The expression level of each lncRNA. Only the lncRNAs with significant changes in their expression between the control and BDL tissues were selected (see Materials and Methods for details). ^∗^*p* < 0.05, ^∗∗^*p* < 0.005, and ^∗∗∗^*p* < 0.0005 (unpaired two-tailed *t*-tests). (b and c) Genomic information for (b) Gas5 and (c) Zfas1 in both the mouse and human genomes. This information was obtained from the UCSC Genome Browser (https://genome.ucsc.edu/). The blue, green, and red colors represent protein-coding, lncRNA, and siRNA genes, respectively.

Previous studies suggest that lncRNAs are involved in the pathogenesis of a diverse range of neuronal diseases. Our expression profiling of the cortex tissues from a BDL model also suggests that many lncRNAs are differentially expressed during hyperammonemia (Supplementary Table S1). We next applied a stringent set of identification criteria and obtained eight differentially expressed lncRNAs likely associated with hepatic encephalopathy in BDL mouse brain tissues (Figure 4(a)). We then validated which of the lncRNAs could play a role in the pathogenesis of this condition by evaluating the documented roles of these transcripts in other diseases. Interestingly, the suppression of lncRNA GAS5 expression attenuated hydrogen peroxide-induced damage in retinal ganglion cells, and the inhibition of lncRNA ZFAS1 expression suppressed hippocampal neuronal apoptosis [29]. Moreover, ZFAS1 silencing also decreased p53 expression [30]. Evaluation of the genomic information revealed that Gas5 was transcribed in the opposite direction to its neighboring gene, Zbtb37, both in mice and humans (Figure 4(b)) and that Zfas1 was the antisense transcript to its neighboring Znfx1 gene in mice and humans (Figure 4(c)). Based on this information, we selected the two lncRNAs and attempted to evaluate whether these lncRNAs could play essential roles in our BDL model.

### 3.5. ZFAS1 and GAS5 Regulate Ammonium Chloride (NH_4_Cl)-Induced Neuronal Apoptosis

It is known that the cells exposed to NH_4_Cl metabolize this molecule into ammonia [31]. The SH-SY5Y cells were differentiated into neurons, treated with siRNA against each lncRNA as well as NH_4_Cl, and then used to investigate the role of these lncRNAs during hyperammonemia-induced apoptosis (see Materials and Methods). ZFAS1 and GAS5 were silenced using two independent siRNAs, and the knockdown efficiency of these constructs was confirmed by qRT-PCR (Figure S4A). The differentiation of SH-SY5Y was confirmed by staining the cells with MAP2 which is abundantly expressed in neuronal cells (Figure S4B), and NH_4_Cl-induced apoptosis was achieved using a 30 mM concentration as identified in our optimization assays (Figure S4C).

**Figure 5:**
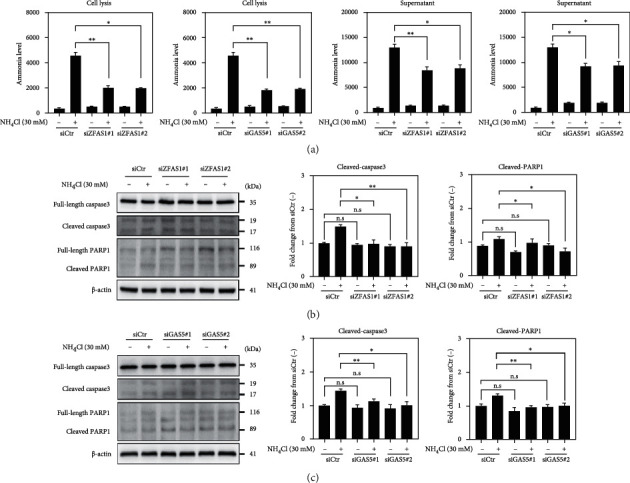
ZFAS1 and GAS5 modulate ammonia metabolism and apoptosis in neurons in response to hyperammonemia. (a) Measurement of ammonia levels in the cell lysates and supernatants of SH-SY5Y cells treated with NH_4_Cl. (b) Western blot showing the protein levels of the apoptotic markers, cleaved-caspase-3 and cleaved-PARP1, following ZFAS1 knockdown in SH-SY5Y cells treated with NH_4_Cl. The level of each protein was normalized against that of *β*-actin. The levels of cleaved proteins were normalized against their full-length protein levels. (c) Western blot showing the protein levels of the apoptotic markers, cleaved-caspase-3 and cleaved-PARP1, following GAS5 knockdown in SH-SY5Y cells treated with NH_4_Cl. The level of each protein was normalized against that of *β*-actin. The levels of cleaved proteins were normalized against their full-length protein levels. Error bars represent the S.E.M. from three independent experiments. ^∗^*p* < 0.05, ^∗∗^*p* < 0.01, and ^∗∗∗^*p* < 0.001 (unpaired two-tailed *t*-tests with Welch's correction).

Then, we evaluated the impact of these lncRNAs on ammonia metabolism by measuring the ammonia levels in the cell lysate and supernatant following siRNA and NH_4_Cl treatment (Figure 5(a)). Ammonia levels in both the supernatant and cell lysates were significantly increased in the NH_4_Cl-treated control neurons compared with the untreated control. However, the increase in intracellular ammonia level was decreased in response to ZFAS1 or GAS5 silencing. This was also true for the supernatant samples. These results suggest that ZFAS1 and GAS5 are involved in the ammonia metabolism of these cells.

Given the demonstrated increase in p53 expression and activation in the cortical tissues of BDL mice, we hypothesized that the p53 pathway was directly implicated in neuronal cell death observed in BDL tissues (Figures 2 and 3). We tested this hypothesis by measuring the expression and activation of the apoptosis-related proteins, cleaved-caspase-3 and cleaved-poly[ADP-ribose] polymerase 1 (PARP-1), under NH_4_Cl-induced hyperammonemia (Figures 5(b) and 5(c)). The expression of cleaved form of the proteins was significantly increased in the NH_4_Cl-treated control neurons compared with the untreated neurons. However, this change in expression was not observed following ZFAS1 or GAS5 knockdown in NH_4_Cl-treated cells. In addition, knockdown of either lncRNA did not alter the basal protein expression of the untreated neurons. Taken together, our results suggest that both ZFAS1 and GAS5 exert proapoptotic effects during NH_4_Cl-induced hyperammonemia.

### 3.6. ZFAS1 and GAS5 Enhance Neurite Complexity during Neuronal Damage

**Figure 6:**
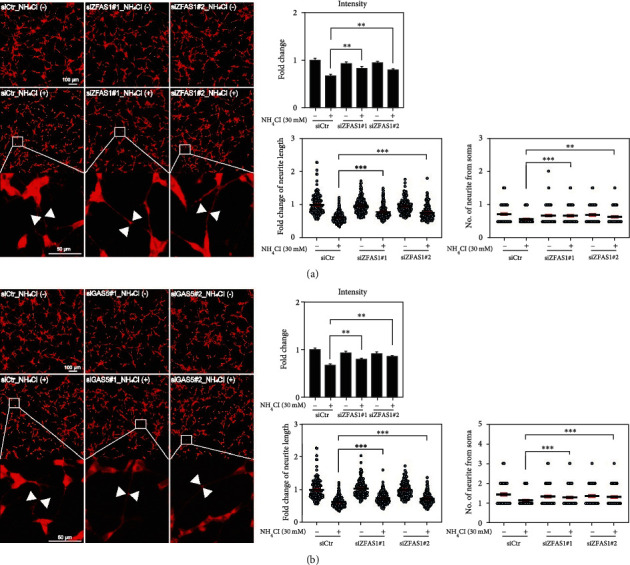
ZFAS1 and GAS5 regulate neuronal complexity during hyperammonemia. (a) Fluorescent images of SH-SY5Y cells treated with NH_4_Cl following ZFAS1 knockdown. Scale bars: 100 *μ*m and 50 *μ*m. More than 400 cells per group were analyzed for neurite length and the number of neurites from the soma. (b) Fluorescent images of SH-SY5Y cells treated with NH_4_Cl following GAS5 knockdown. Scale bars: 100 *μ*m and 50 *μ*m. More than 400 cells per group were analyzed for neurite length and the number of neurites from the soma. Error bars represent the S.E.M. from three independent experiments. ^∗^*p* < 0.05, ^∗∗^*p* < 0.01, and ^∗∗∗^*p* < 0.001 (unpaired two-tailed *t*-tests with Welch's correction).

We confirmed the decrease in Map2 expression during hyperammonemia in our model, which suggests that there is a significant dysregulation of neural structure in these animals (Figures 1(c) and 1(d)). Accordingly, we next evaluated the function of the two critical lncRNAs identified in this study using the fluorescent analysis of both neurite complexity and neurite outgrowth in BDL tissues (Figures 6(a) and 6(b)). Fluorescence intensity was significantly reduced in NH_4_Cl-treated neurons with neuronal damage compared to the untreated control. However, these decreased intensities were partially restored when ZFAS1 and GAS5 expression was inhibited using siRNA (Figures 6(a) and 6(b), left panels). Neuronal complexity, including neurite length and the number of neurites from the soma, decreased in NH_4_Cl-treated neurons. In contrast, knockdown of ZFAS1 and GAS5 improved neuronal complexity in spite of the presence of NH_4_Cl (Figures 6(a) and 6(b), right panel). In addition, the complexity of the untreated neurons was not affected by the inhibition of either lncRNA. These data suggest that the suppression of the lncRNAs, ZFAS1 and GAS5, could improve neuronal connectivity during hyperammonemia-induced neuronal damage.

## 4. Discussion

Hepatic encephalopathy is characterized by aberrant increases in ammonia levels in the blood and brain, which ultimately leads to neurological impairment [32] and neuronal cell death [9]. However, the molecular mechanisms underlying hepatic encephalopathy are not completely understood. In this study, we used a mouse model of BDL to induce hepatic failure and mimic hyperammonemia to investigate hepatic encephalopathy. Our *in vivo* experiments demonstrated that BDL mice present with an increase in ammonia level, cell death, and neuronal damage in the cerebral cortex. In addition, the transcriptomic analysis revealed that BDL mice experience an increase in ammonia-related Akt/S6K/p53 signaling, IL-6/JAK/Stat3 signaling, and TNF-*α* signaling while undergoing a decrease in neurogenesis and cellular morphogenesis in the cerebral cortex. Importantly, we identified several candidate lncRNAs, including Zfas1 and Gas5, which showed differential expression in the brain of normal and BDL mice. Further investigation of the functions of these lncRNAs (ZFAS1 and GAS5) revealed that both transcripts were implicated in the modulation of extracellular and intracellular ammonia levels and apoptosis in neuronal cell culture. In addition, their deficiency led to changes in the neuronal structure, including differences in the number and length of neurites. Thus, ZFAS1 and GAS5 lncRNAs may contribute to hyperammonemia-induced neuronal injury and cell death.

Our RNA sequencing data showed that the expression of ZBTB37 and ZNFX1, the neighboring genes of GAS5 and ZFAS1, did not undergo any marked changes in terms of transcript level (data not shown). Therefore, these lncRNAs and their neighboring genes are not likely to have a regulatory relationship. Remarkably, the expression levels of both of these lncRNAs were shown to increase in ischemic stroke and myocardial infarction in a previous analysis, suggesting their involvement in diverse diseases [15]. Interestingly, lncRNAs GAS5 and ZFAS1 contain snoRNA genes within their introns, suggesting that these lncRNAs may operate simultaneously with their snoRNAs.

The leading cause of neuronal cell death and structural abnormalities in hepatic encephalopathy is probably hyperammonemia [9]. According to the previous studies, hyperammonemia induces apoptosis in primary hepatocytes, HepG2 cells, and cortical neurons [9, 33]. In addition, it has been reported that NH_4_Cl treatment induces the expression of apoptotic markers, such as caspase-3 and cytochrome C, *in vitro* [33]. Ammonia neurotoxicity has also been linked to increased cytoplasmic p53 expression in the brain [34], and acute ammonia increases apoptosis via enhanced p53, Bcl-2-associated X protein (BAX), and caspase family (caspase-8, 9, and 3) expression in epithelial cells [26]. However, the evidence that ammonia-induced p53, Akt, and mTORC1 signaling pathways are involved in neuronal cell death lacks to elucidate until now. It is known that one of the primary functions of p53 is the regulation of apoptosis [35]. The p53 is associated with Akt and mTORC1 signaling [36, 37], and ammonium promotes Akt (S473)-dependent activation of mTORC1 signaling [27]. In the present study, we demonstrated that the BDL model experiences increased Akt (S473), mTORC1 (S6K), and p53 signaling under hyperammonemia conditions. Thus, the interplay between Akt/mTORC1 and p53 signaling might be involved in hyperammonemia-induced apoptosis.

In addition, p53 is closely associated with TNF-*α*-induced apoptosis [38]. A previous study demonstrated that p53 mediates TNF-*α*-induced apoptotic cell death and overexpression of p53 results in an increase of TNF-*α*-induced apoptotic cell death in oligodendrocytes [38]. Additionally, TNF-*α* induces ammonia transport from the circulatory system to brain regions and plays an important role in neuronal cell death [39, 40]. Importantly, hepatic encephalopathy patients with chronic liver failure display increased level of circulating TNF-*α*, and TNF-*α* and ammonia show a positive correlation in the patients with hepatic encephalopathy [41]. Furthermore, TNF-*α* levels correlate with the severity of hepatic encephalopathy [41]. Also, the activation of IL-6/JAK/STAT signaling is observed in steatohepatitis and hepatocellular carcinoma [42]. In clinical study, circulating IL-6 efficiently correlates with ammonia level in patients with minimal hepatic encephalopathy [43]. Similar to TNF-*α*, IL-6 was also correlated with the severity of hepatic encephalopathy [43]. In the present study, we also observed increased levels of TNF-*α* and IL-6/Stat3 signaling in the cerebral cortex, indicating that our model is consistent with the hepatic encephalopathy phenotype. Because cell death can be caused by a various range of mechanisms, however, there could be another pathway involved in the cell death in hepatic encephalopathy than the identified mechanism above.

**Figure 7:**
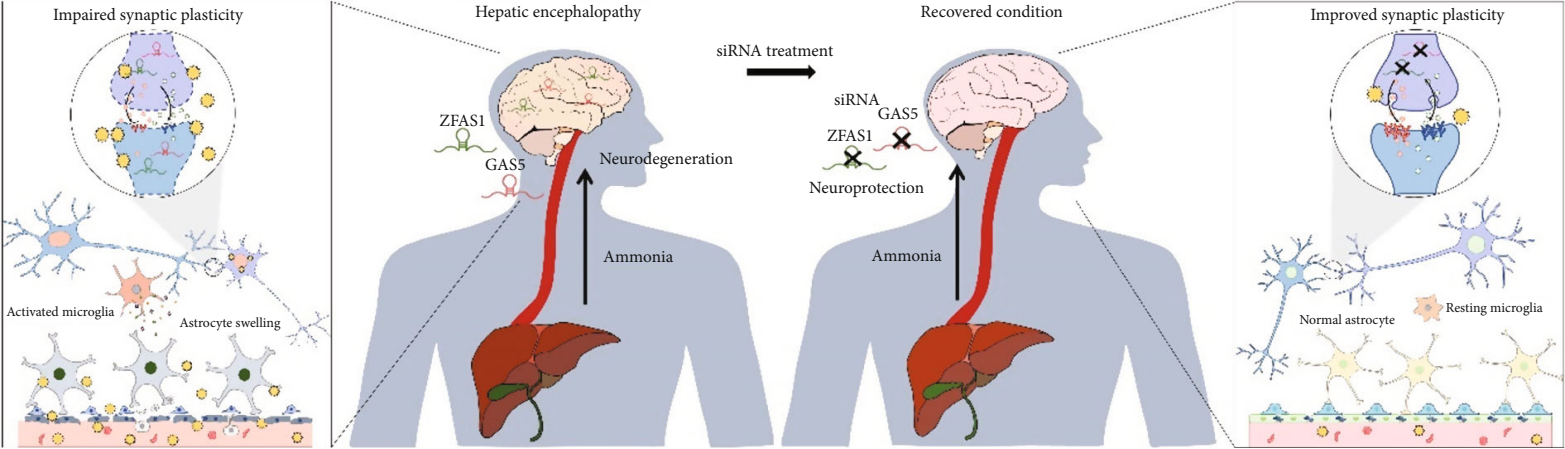
Strategies for modulation of lncRNAs ZFAS1 and GAS5 to control hepatic encephalopathy.

Hyperammonemia is closely associated with neuronal cell death, the disruption of neuronal morphology, synaptic vesicle release, and long-term synaptic change [7, 31]. Patients with minimal hepatic encephalopathy have shown impaired learning and memory, accompanied by changes in the hippocampal structure and function [44]. This was consistent with our mouse and cell culture experiments, which demonstrated abnormal neuronal structure (reduced levels of NeuN and MAP2) in response to hepatic encephalopathy, although there is no significant alteration of Psd-95 level. In addition, treatment with ammonia-reducing substances decreased hepatic encephalopathy symptoms in patients with cirrhosis [45]. Moreover, ameliorating ammonia levels in blood prevents damage and apoptosis in the hepatocytes of an acute hepatic failure model [46]. Therefore, ammonia-lowering strategies have been proposed as a clinical intervention for hepatic encephalopathy [47]. Because our results linked GAS5 and ZFAS1 to the ammonia metabolism of the cortical tissues, we propose that the modulation of these lncRNAs could be a promising strategy to control hepatic encephalopathy (Figure 7).

## Data Availability

The data sets used and/or analyzed during the current study are available from the corresponding author on reasonable request.

## Abbreviations

BAD: BCL2-associated agonist of cell death
lncRNAs: Long noncoding RNAs
FBS: Fetal bovine serum
FPKM: Fragments per kilobase of transcript per million mapped reads
MSigDB: Molecular Signatures Database
SDS: Sodium dodecyl sulfate
PVDF: Polyvinylidene difluoride
S6K: p70S6 kinase.

## Supplementary Materials

Supplementary 1 Supplementary Figure 1: experimental scheme and reagent information. (A) Experimental scheme describing our investigation of the functions of lncRNAs during neuronal apoptosis and neuronal complexity in response to NH_4_Cl using SH-SY5Y cells. (B) Sequence information for the PCR primers used in this study. (C) The sequences of the siRNAs used in the knockdown experiments. (D) Description of the antibodies used in this study. Supplementary Figure 2: immunohistochemistry for Map2 in the cerebral cortex 2 weeks after BDL. Supplementary Figure 3: IL-6 concentration (pg/ml) in the cerebral cortex 2 weeks after BDL surgery. Supplementary Figure 4: the efficiency of the lncRNA-targeting siRNAs, the confirmation of neuronal differentiation of SH-SY5Y cells, and the optimization of the NH_4_Cl concentrations. (A) The efficiency of the lncRNA-targeting siRNAs in SH-SY5Y cells. Error bars represent the S.E.M. from three independent experiments. (B) The staining of SH-SY5Y cells with MAP2 confirmed neuronal differentiation. At five days of retinoic acid treatment, the mature neuron-like dendritic formation of SH-SY5Y cells was observed. (C) Optimization of the NH_4_Cl concentrations used in this study. Cell viability was measured using the WST assay 24 h after exposure to varying (0 to 40 mM) concentrations of NH_4_Cl. Error bars represent the S.E.M. from six independent experiments.

Supplementary 2 Supplementary Table 1: expression levels of the lncRNAs from the control and BDL mice. lncRNA expression was evaluated using two different analysis algorithms (see Materials and Methods for details).
