# Supplementary material for: Long Noncoding RNAs Regulate Hyperammonemia-Induced Neuronal Damage in Hepatic Encephalopathy
Source: Oxid Med Cell Longev. 2022 Feb 21;2022:7628522. doi: 10.1155/2022/7628522 (PMC9021992; doi:10.1155/2022/7628522)
Supplement: Supplementary 1 — Supplementary Figure 1: experimental scheme and reagent information. (A) Experimental scheme describing our investigation of the functions of lncRNAs during neuronal apoptosis and neuronal complexity in response to NH4Cl using SH-SY5Y cells. (B) Sequence information for the PCR primers used in this study. (C) The sequences of the siRNAs used in the knockdown experiments. (D) Description of the antibodies used in this study. Supplementary Figure 2: immunohistochemistry for Map2 in the cerebral cortex 2 weeks after BDL. Supplementary Figure 3: IL-6 concentration (pg/ml) in the cerebral cortex 2 weeks after BDL surgery. Supplementary Figure 4: the efficiency of the lncRNA-targeting siRNAs, the confirmation of neuronal differentiation of SH-SY5Y cells, and the optimization of the NH4Cl concentrations. (A) The efficiency of the lncRNA-targeting siRNAs in SH-SY5Y cells. Error bars represent the S.E.M. from three independent experiments. (B) The staining of SH-SY5Y cells with MAP2 confirmed neuronal differentiation. At five days of retinoic acid treatment, the mature neuron-like dendritic formation of SH-SY5Y cells was observed. (C) Optimization of the NH4Cl concentrations used in this study. Cell viability was measured using the WST assay 24 h after exposure to varying (0 to 40 mM) concentrations of NH4Cl. Error bars represent the S.E.M. from six independent experiments. [file 7628522.f1.docx]

**Supplementary materials**


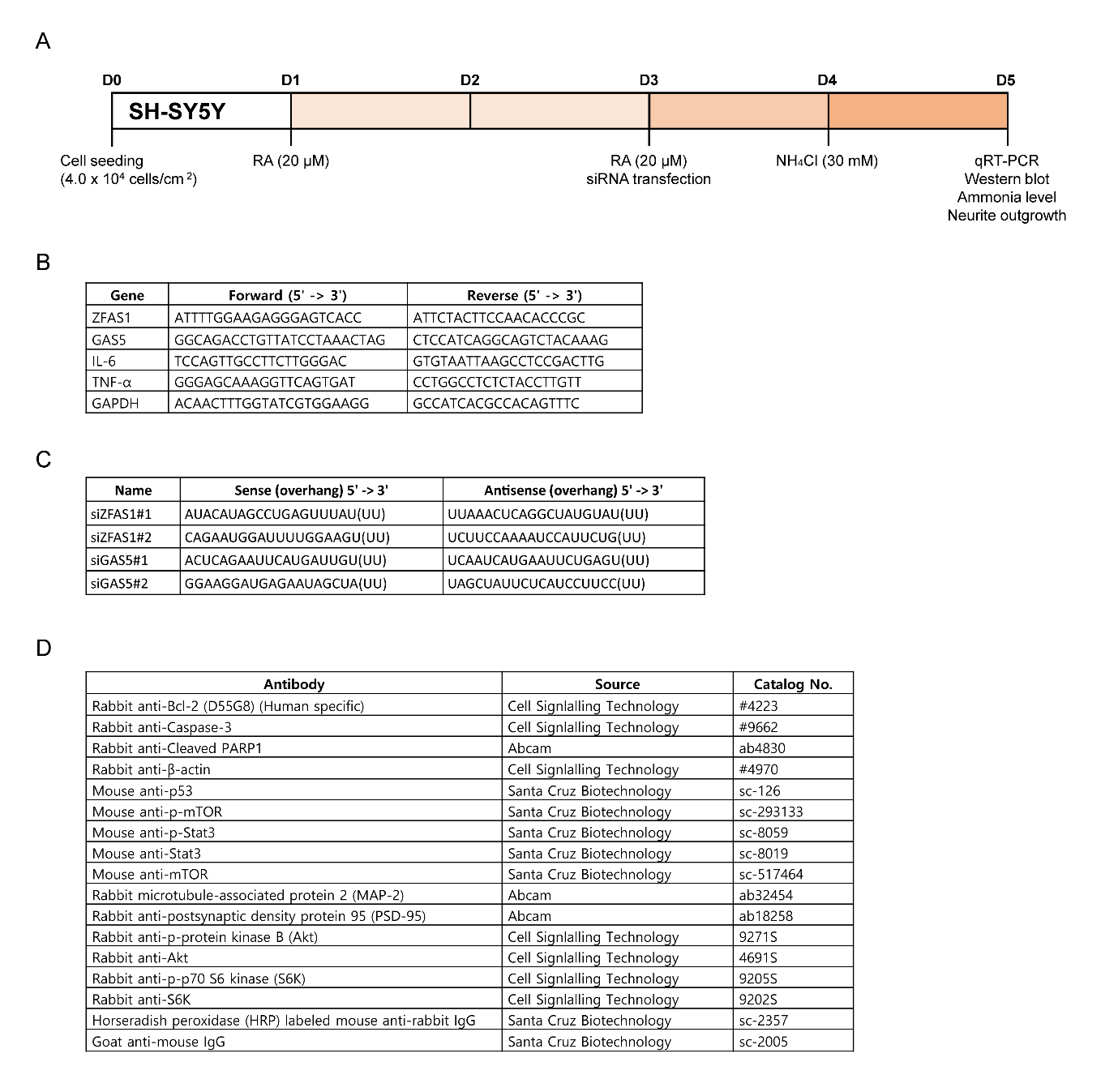


**Supplementary Figure 1.** Experimental scheme and reagent information. (A) Experimental scheme describing our investigation of the functions of lncRNAs during neuronal apoptosis and neuronal complexity in response to NH_4_Cl using SH-SY5Y cells. (B) Sequence information for the PCR primers used in this study. (C) The sequences of the siRNAs used in the knockdown experiments. (D) Description of the antibodies used in this study.


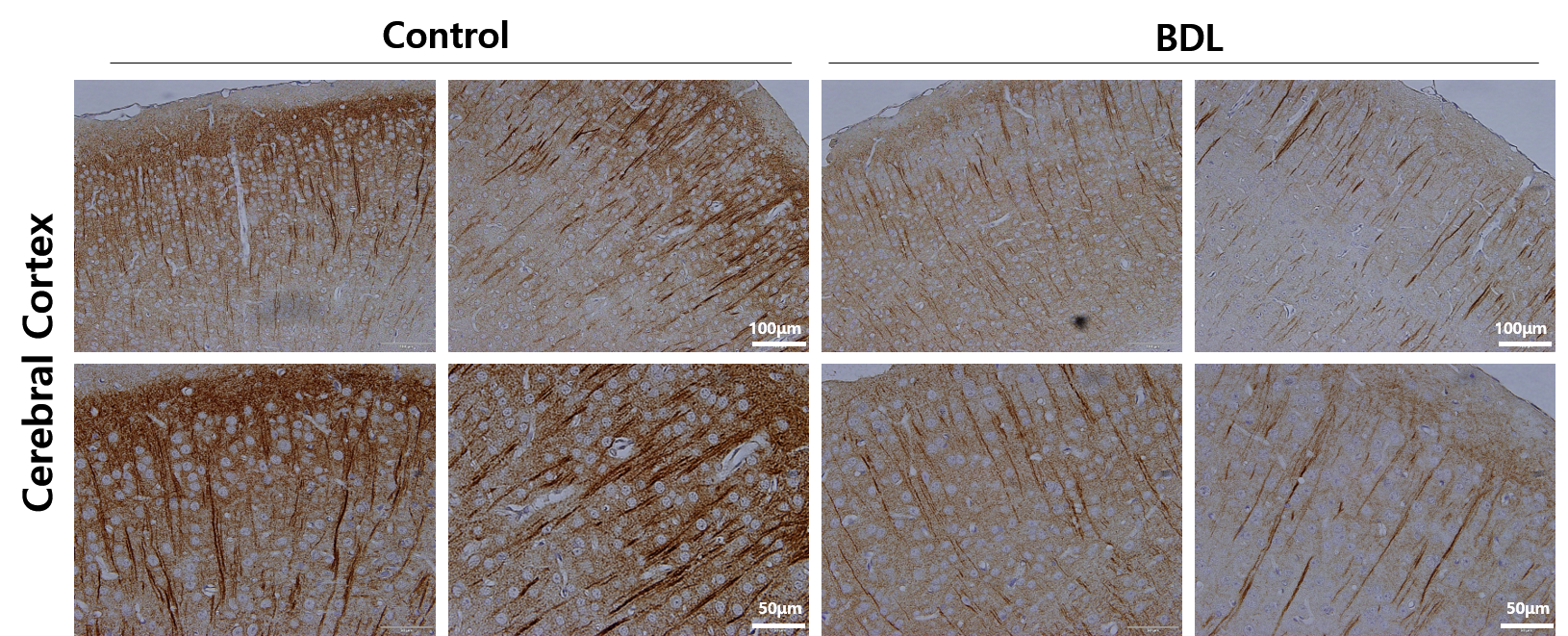


**Supplementary Figure 2.** Immunohistochemistry for Map-2 in the cerebral cortex 2 weeks after BDL.


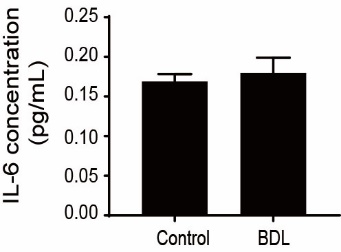


**Supplementary Figure 3.** IL-6 concentration (pg/mL) in the cerebral cortex 2 weeks after BDL surgery.


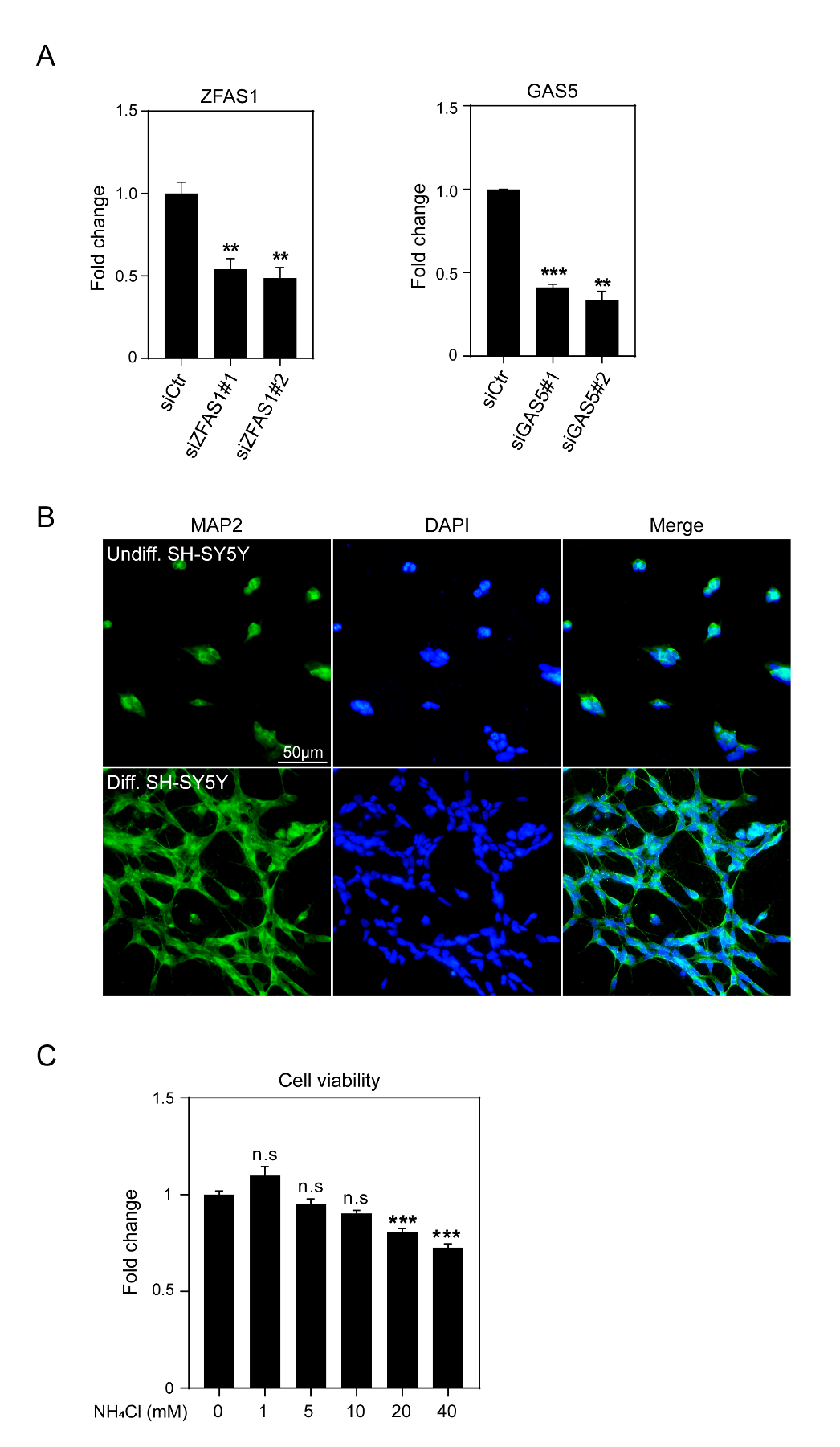


**Supplementary Figure 4.** The efficiency of the lncRNA-targeting siRNAs, the confirmation of neuronal differentiation of SH-SY5Y cells, and the optimization of the NH_4_Cl concentrations. (A) The efficiency of the lncRNA-targeting siRNAs in SH-SY5Y cells. Error bars represent the S.E.M. from three independent experiments. (B) The staining of SH-SY5Y cells with MAP2 confirmed neuronal differentiation. At five days of retinoic acid treatment, the mature neuron-like dendritic formation of SH-SY5Y cells was observed. (C) Optimization of the NH_4_Cl concentrations used in this study. Cell viability was measured using the WST assay 24 h after exposure to varying (0 to 40 mM) concentrations of NH_4_Cl. Error bars represent the S.E.M. from six independent experiments.

**Supplementary Table 1.** Expression levels of the lncRNAs from control and BDL mice. lncRNA expression was evaluated using two different analysis algorithms. See methods for details.
