# Supplementary material for: Long Noncoding RNAs Regulate Hyperammonemia-Induced Neuronal Damage in Hepatic Encephalopathy
Source: Oxid Med Cell Longev. 2022 Feb 21;2022:7628522. doi: 10.1155/2022/7628522 (PMC9021992; doi:10.1155/2022/7628522)
Supplement: Supplementary 2 — Supplementary Table 1: expression levels of the lncRNAs from the control and BDL mice. lncRNA expression was evaluated using two different analysis algorithms (see Materials and Methods for details). [file 7628522.f2.pdf]

						Cuffnorm				Salmon			
Gene ID	Gene class	Gene name	Description	Avr	Min	Con.Avr	BDL.Avr	Log2fold	T-test	logCPM	logFC	P-Value	Direction
ENSMUSG00000105265.4	processed_transcript	Sox2ot	SOX2 overlapping transcript (non-protein	11.2	7.5	14.3	8.1	-0.81	**0.000**	3.95	-0.80	**0.000**	Decrease
ENSMUSG00000086370.8	lincRNA	Ftx	Ftx transcript, Xist regulator (non-protein c	14.5	12.5	12.9	16.0	0.31	**0.005**	4.96	0.29	**0.006**	Increase
ENSMUSG00000082286.10	transcribed_unprocessed_pse	Pisd-ps1	phosphatidylserine decarboxylase, pseud	152.1	125.2	131.0	173.1	0.40	**0.005**	6.89	0.37	**0.001**	Increase
ENSMUSG00000053332.13	processed_transcript	Gas5	growth arrest specific 5 [Source:MGI Sym	76.3	63.9	67.5	85.1	0.33	**0.008**	4.63	0.32	**0.007**	Increase
ENSMUSG00000102101.1	antisense	Zbtb11os1	zinc finger and BTB domain containing 11,	1.9	1.0	1.3	2.5	0.98	**0.011**	0.79	0.85	**0.004**	Increase
ENSMUSG00000097767.8	lincRNA	Miat	myocardial infarction associated transcrip	56.2	47.7	50.6	61.8	0.29	**0.019**	8.49	0.27	**0.007**	Increase
ENSMUSG00000074578.13	processed_transcript	Zfas1	zinc finger, NFX1-type containing 1, antise	17.1	11.8	13.9	20.3	0.55	**0.021**	2.88	0.55	**0.003**	Increase
ENSMUSG00000085396.7	processed_transcript	Firre	functional intergenic repeating RNA eleme	6.8	5.6	5.9	7.7	0.37	**0.033**	4.97	0.33	**0.008**	Increase
ENSMUSG00000087590.2	lincRNA	Epb41l4aos	erythrocyte membrane protein band 4.1 lik	7.6	5.9	6.3	8.8	0.48	**0.003**	0.77	0.40	0.148	Increase
ENSMUSG00000072653.13	transcribed_unprocessed_pse	Zfp783	zinc finger protein 783 [Source:MGI Symb	1.6	1.5	1.5	1.7	0.16	**0.005**	1.91	-0.15	0.561	
ENSMUSG00000023795.16	transcribed_unprocessed_pse	Pisd-ps2	phosphatidylserine decarboxylase, pseud	22.6	17.9	19.6	25.7	0.39	**0.019**	4.82	0.24	0.110	Increase
ENSMUSG00000085148.1	processed_transcript	Mir22hg	Mir22 host gene (non-protein coding) [Sou	14.4	12.3	12.8	16.0	0.32	**0.023**	4.07	0.30	**0.031**	Increase
ENSMUSG00000097320.8	antisense	Tmem147os	transmembrane protein 147, opposite stra	7.1	6.4	6.7	7.5	0.18	**0.029**	2.57	0.18	0.291	Increase
ENSMUSG00000097164.2	bidirectional_promoter_lncRNA	Cep83os	centrosomal protein 83, opposite strand [S	4.8	4.2	4.4	5.2	0.23	**0.033**	4.46	0.20	0.084	Increase
ENSMUSG00000046808.17	polymorphic_pseudogene	Atp10d	ATPase, class V, type 10D [Source:MGI S	1.7	1.1	1.3	2.1	0.72	**0.035**	2.22	0.51	0.103	Increase
ENSMUSG00000085385.7	processed_transcript	Snhg17	small nucleolar RNA host gene 17 [Source	5.7	3.6	4.6	6.8	0.56	**0.036**	2.44	0.69	**0.001**	Increase
ENSMUSG00000096780.7	transcribed_unprocessed_pse	Tmem181b-ps	transmembrane protein 181B, pseudogene	49.9	38.2	44.4	55.4	0.32	**0.050**	6.92	0.27	**0.016**	Increase
ENSMUSG00000097059.1	lincRNA	Fam120aos	family with sequence similarity 120A, oppo	3.6	3.1	3.3	3.9	0.25	0.051	2.31	0.23	0.235	Increase
ENSMUSG00000100252.6	lincRNA	Mir124-2hg	Mir124-2 host gene (non-protein coding) [S	30.2	26.8	28.2	32.1	0.19	0.054	5.52	0.20	**0.050**	Increase
ENSMUSG00000021268.17	lincRNA	Meg3	maternally expressed 3 [Source:MGI Sym	311.9	262.2	286.6	337.1	0.23	0.069	10.57	0.22	**0.033**	Increase
ENSMUSG00000086290.8	lincRNA	Snhg12	small nucleolar RNA host gene 12 [Source	20.8	14.5	17.8	23.9	0.43	0.070	3.03	0.37	**0.038**	Increase
ENSMUSG00000084880.1	antisense	Tomm6os	translocase of outer mitochondrial membra	1.5	1.1	1.3	1.8	0.44	0.071	1.65	0.40	0.108	Increase
ENSMUSG00000044471.12	lincRNA	Lncpint	long non-protein coding RNA, Trp53 induc	26.1	21.0	23.6	28.7	0.28	0.085	4.61	0.28	0.074	Increase
ENSMUSG00000083844.8	transcribed_unprocessed_pse	Ube2d-ps	ubiquitin-conjugating enzyme E2D, pseudo	8.5	7.9	8.1	8.9	0.14	0.090	3.31	0.12	0.398	Increase
ENSMUSG00000089281.1	scaRNA	Scarna6	small Cajal body-specific RNA 6 [Source:M	235.6	198.9	212.1	259.1	0.29	0.091	4.17	0.26	0.070	Increase
ENSMUSG00000084799.7	lincRNA	Ino80dos	INO80 complex subunit D, opposite strand	3.4	2.0	2.8	4.0	0.50	0.097	0.94	0.58	**0.048**	Increase
ENSMUSG00000060424.14	lincRNA	Pantr1	POU domain, class 3, transcription factor	27.9	17.0	32.2	23.7	-0.44	0.100	3.69	-0.43	**0.022**	Decrease
ENSMUSG00000097451.10	processed_transcript	Rian	RNA imprinted and accumulated in nucleu	522.4	485.6	541.7	503.1	-0.11	0.114	8.98	-0.11	0.244	Decrease
ENSMUSG00000022639.14	lincRNA	Dubr	Dppa2 upstream binding RNA [Source:MG	3.8	3.1	4.3	3.4	-0.32	0.122	3.61	-0.20	0.141	Decrease
ENSMUSG00000063171.4	transcribed_processed_pseud	Rps4l	ribosomal protein S4-like [Source:MGI Sy	10.4	7.5	8.4	12.3	0.56	0.123	2.53	0.53	**0.036**	Increase
ENSMUSG00000031838.8	processed_transcript	Ifi30	interferon gamma inducible protein 30 [Sou	1.8	0.8	1.3	2.3	0.80	0.124	0.40	0.65	0.104	Increase
ENSMUSG00000086212.7	lincRNA	Mkln1os	muskelin 1, intracellular mediator containin	1.9	1.3	1.6	2.3	0.50	0.125	0.59	0.54	0.089	Increase
ENSMUSG00000006395.16	polymorphic_pseudogene	Hyi	hydroxypyruvate isomerase (putative) [So	3.8	3.2	3.4	4.2	0.30	0.133	1.24	0.30	0.241	Increase
ENSMUSG00000065145.1	misc_RNA	Vaultrc5	vault RNA component 5 [Source:MGI Sym	195.5	139.8	217.3	173.6	-0.32	0.154	0.60	-0.18	0.563	Decrease
ENSMUSG00000086859.3	lincRNA	Snhg20	small nucleolar RNA host gene 20 [Source	27.1	21.7	24.6	29.7	0.27	0.169	3.28	0.27	0.123	Increase
ENSMUSG00000085028.2	transcribed_unitary_pseudoge	Slc2a4rg-ps	Slc2a4 regulator, pseudogene [Source:MG	2.8	2.2	3.1	2.6	-0.21	0.175	2.71	-0.23	0.208	Decrease
ENSMUSG00000085241.7	processed_transcript	Snhg3	small nucleolar RNA host gene 3 [Source:	4.2	2.9	3.5	4.9	0.48	0.188	0.58	0.45	0.178	Increase
ENSMUSG00000090063.6	antisense	Dlx6os1	distal-less homeobox 6, opposite strand 1	4.8	4.0	5.1	4.5	-0.17	0.192	3.96	-0.18	0.187	Decrease
ENSMUSG00000093452.4	antisense	Zfhx2os	zinc finger homeobox 2, opposite strand [S	1.4	1.0	1.3	1.6	0.34	0.193	2.27	0.32	0.179	Increase
ENSMUSG00000097814.5	antisense	Panct2	pluripotency-associated noncoding transc	1.8	1.5	2.0	1.7	-0.21	0.212	2.83	-0.26	0.152	Decrease
ENSMUSG00000072692.7	transcribed_processed_pseud	Rpl37rt	ribosomal protein L37, retrotransposed [S	4.7	1.8	3.5	5.9	0.76	0.212	3.83	0.14	0.367	Increase
ENSMUSG00000103081.2	polymorphic_pseudogene	Pcdhgb8	protocadherin gamma subfamily B, 8 [Sou	1.4	1.0	1.6	1.3	-0.29	0.216	2.04	-0.28	0.218	Decrease

ENSMUSG00000099784.1	lincRNA	Dalir	DNMT1 associated long intergenic non-cc	1.4	0.7	1.5	1.2	-0.42	0.218	1.07	-0.44	0.172	Decrease
ENSMUSG00000085208.3	antisense	Brip1os	BRCA1 interacting protein C-terminal helic	2.5	1.8	2.7	2.3	-0.25	0.223	3.24	-0.11	0.530	Decrease
ENSMUSG00000097316.2	antisense	CAAA01201205.3	predicted gene 10516 [Source:MGI Symb	1.3	0.9	1.2	1.5	0.31	0.247	1.78	0.27	0.237	Increase
ENSMUSG00000074876.5	processed_transcript	Spata5l1	spermatogenesis associated 5-like 1 [Sou	1.4	1.2	1.6	1.3	-0.23	0.253	1.53	-0.23	0.353	Decrease
ENSMUSG00000108414.1	processed_transcript	Snhg1	small nucleolar RNA host gene 1 [Source:	8.8	6.2	8.0	9.7	0.28	0.271	1.72	0.22	0.370	Increase
ENSMUSG00000096954.1	lincRNA	Gdap10	ganglioside-induced differentiation-associa	1.6	1.5	1.5	1.7	0.13	0.283	2.50	0.18	0.336	Increase
ENSMUSG00000105361.1	lincRNA	Yam1	cDNA sequence AY036118 [Source:MGI S	290.2	75.1	120.1	460.4	1.94	0.308	7.45	1.96	**0.007**	Increase
ENSMUSG00000024845.17	processed_transcript	Tmem134	transmembrane protein 134 [Source:MGI	3.0	2.3	2.8	3.2	0.19	0.308	3.18	0.18	0.267	Increase
ENSMUSG00000097589.9	processed_transcript	Dleu2	deleted in lymphocytic leukemia, 2 [Source	7.2	6.3	7.7	6.7	-0.22	0.317	3.22	-0.26	0.298	Decrease
ENSMUSG00000106106.2	rRNA	Rn18s-rs5	18s RNA, related sequence 5	91.7	69.4	76.4	106.9	0.49	0.319	0.70	0.56	0.143	Increase
ENSMUSG00000115420.1	lincRNA	Rmrp	RNA component of mitochondrial RNAase	1661.0	1180.4	1544.5	1777.5	0.20	0.322	7.15	0.19	0.300	Increase
ENSMUSG00000063714.15	antisense	Sp3os	trans-acting transcription factor 3, opposit	5.1	3.2	4.1	6.0	0.55	0.325	0.97	0.57	0.117	Increase
ENSMUSG00000097039.8	lincRNA	Pvt1	Pvt1 oncogene [Source:MGI Symbol;Acc:	6.7	5.5	7.1	6.3	-0.17	0.328	3.12	-0.22	0.250	Decrease
ENSMUSG00000080059.4	processed_pseudogene	Rps19-ps3	ribosomal protein S19, pseudogene 3 [Sou	4.2	2.4	3.6	4.7	0.41	0.328	0.30	-0.13	0.709	
ENSMUSG00000029447.11	processed_transcript	Cct6a	chaperonin containing Tcp1, subunit 6a (z	51.0	47.7	52.2	49.9	-0.06	0.346	6.40	-0.06	0.556	Decrease
ENSMUSG00000041596.12	transcribed_unprocessed_pse	Nlrp5-ps	NLR family, pyrin domain containing 5, pse	7.9	5.4	9.2	6.7	-0.46	0.365	2.99	-0.49	0.090	Decrease
ENSMUSG00000086171.7	antisense	Pcsk2os1	proprotein convertase subtilisin/kexin type	2.5	1.7	2.7	2.2	-0.27	0.375	2.10	-0.04	0.867	Decrease
ENSMUSG00000064043.13	processed_transcript	Trerf1	transcriptional regulating factor 1 [Source:	5.4	4.9	5.2	5.6	0.11	0.391	4.82	0.09	0.475	Increase
ENSMUSG00000097195.9	processed_transcript	Snhg5	small nucleolar RNA host gene 5 [Source:	5.1	4.2	4.9	5.3	0.13	0.394	1.73	0.05	0.810	Increase
ENSMUSG00000085837.8	antisense	Kcnmb4os2	potassium large conductance calcium-act	1.1	0.8	1.0	1.1	0.19	0.400	0.01	0.08	0.834	Increase
ENSMUSG00000052248.15	antisense	Zeb2os	zinc finger E-box binding homeobox 2, op	2.0	0.8	2.2	1.8	-0.35	0.411	0.87	-0.25	0.487	Decrease
ENSMUSG00000083274.1	processed_pseudogene	Zcchc9-ps	zinc finger, CCHC domain containing 9, ps	1.9	0.5	1.7	2.2	0.35	0.418	0.07	0.38	0.482	Increase
ENSMUSG00000115783.1	lincRNA	Bc1	brain cytoplasmic RNA 1 [Source:MGI Sy	165851.2	146343.0	171528.7	160173.7	-0.10	0.432	10.94	-0.09	0.473	Decrease
ENSMUSG00000098234.7	lincRNA	Snhg6	small nucleolar RNA host gene 6 [Source:	16.6	13.9	15.8	17.4	0.14	0.436	2.41	0.01	0.948	Increase
ENSMUSG00000029386.15	processed_transcript	Tctn2	tectonic family member 2 [Source:MGI Sy	4.5	3.3	4.7	4.3	-0.14	0.459	3.13	-0.17	0.361	Decrease
ENSMUSG00000074346.6	antisense	Kcnd3os	potassium voltage-gated channel, Shal-rel	1.6	0.8	1.2	2.0	0.74	0.460	0.06	0.62	0.256	Increase
ENSMUSG00000088185.1	scaRNA	Scarna2	small Cajal body-specific RNA 2 [Source:M	211.4	194.0	215.4	207.3	-0.06	0.469	5.25	-0.07	0.539	Decrease
ENSMUSG00000107476.2	transcribed_unitary_pseudoge	Zfp862-ps	zinc finger protein 862, pseudogene [Sour	1.6	1.4	1.6	1.5	-0.08	0.496	2.47	-0.15	0.428	Decrease
ENSMUSG00000044081.7	processed_transcript	Zfp85os	zinc finger protein 85, opposite strand [So	1.2	0.9	1.3	1.1	-0.16	0.510	1.03	-0.05	0.862	Decrease
ENSMUSG00000085492.7	processed_transcript	Trmt61b	tRNA methyltransferase 61B [Source:MGI	2.3	1.9	2.3	2.4	0.09	0.522	2.10	0.10	0.610	Increase
ENSMUSG00000106847.1	sense_intronic	Peg13	paternally expressed 13 [Source:MGI Syn	71.7	51.8	74.4	69.1	-0.11	0.544	7.85	-0.12	0.462	Decrease
ENSMUSG00000090386.9	processed_transcript	Mir99ahg	Mir99a and Mirlet7c-1 host gene (non-prot	5.9	4.6	6.3	5.5	-0.18	0.549	3.41	-0.08	0.677	Decrease
ENSMUSG00000097715.2	transcribed_unprocessed_pse	Gpr137b-ps	G protein-coupled receptor 137B, pseudo	3.4	2.6	3.3	3.6	0.14	0.567	2.55	0.08	0.760	Increase
ENSMUSG00000097881.7	lincRNA	Celrr	cerebellum expressed regulatory RNA [So	1.2	0.8	1.1	1.3	0.20	0.569	0.39	0.22	0.510	Increase
ENSMUSG00000063235.16	processed_transcript	Ptpmt1	protein tyrosine phosphatase, mitochondri	4.4	4.0	4.3	4.5	0.06	0.594	3.36	0.04	0.782	Increase
ENSMUSG00000097545.7	lincRNA	Mir124a-1hg	Mir124-1 host gene (non-protein coding) [	42.3	34.6	40.6	43.9	0.11	0.604	6.90	0.10	0.484	Increase
ENSMUSG00000032846.10	processed_transcript	Zswim6	zinc finger SWIM-type containing 6 [Sourc	6.7	5.3	6.3	7.1	0.16	0.605	4.73	0.16	0.410	Increase
ENSMUSG00000086429.9	antisense	Gt(ROSA)26Sor	gene trap ROSA 26, Philippe Soriano [Sou	4.9	3.7	4.8	5.1	0.11	0.612	1.81	0.17	0.489	Increase
ENSMUSG00000032443.16	transcribed_unprocessed_pse	Zcwpw2	zinc finger, CW type with PWWP domain 2	3.4	2.4	3.2	3.6	0.17	0.635	1.08	-0.24	0.531	
ENSMUSG00000092274.2	lincRNA	Neat1	nuclear paraspeckle assembly transcript	12.1	10.3	12.3	11.9	-0.04	0.672	5.82	-0.05	0.648	Decrease
ENSMUSG00000083674.3	transcribed_unitary_pseudoge	Zfp133-ps	zinc finger protein 133, pseudogene [Sour	2.2	1.4	2.0	2.3	0.21	0.687	0.78	0.34	0.260	Increase
ENSMUSG00000101609.2	antisense	Kcnq1ot1	KCNQ1 overlapping transcript 1 [Source:M	4.0	3.2	3.9	4.0	0.05	0.689	8.37	0.03	0.818	Increase
ENSMUSG00000056579.18	antisense	Tug1	taurine upregulated gene 1 [Source:MGI S	21.1	19.1	21.3	20.9	-0.03	0.706	6.37	-0.04	0.715	Decrease
ENSMUSG00000093629.1	antisense	Prox2os	prospero homeobox 2 opposite strand [So	3.5	2.2	3.6	3.5	-0.07	0.707	1.50	0.00	0.988	

ENSMUSG00000104960.1	processed_transcript	Snhg8	small nucleolar RNA host gene 8 [Source:	29.2	22.6	28.4	30.0	0.08	0.749	2.72	0.07	0.739	Increase
ENSMUSG00000084319.3	processed_pseudogene	Tpt1-ps3	tumor protein, translationally-controlled, ps	2.1	1.2	2.1	2.1	-0.04	0.759	4.55	0.00	0.999	Decrease
ENSMUSG00000085436.1	antisense	Zfp335os	zinc finger protein 335, opposite strand [S	1.5	0.9	1.5	1.6	0.06	0.761	0.38	0.14	0.697	Increase
ENSMUSG00000112941.1	lincRNA	CH25-660D15.2	predicted gene, 48623 [Source:MGI Symb	17.6	15.3	17.3	17.9	0.05	0.761	2.14	0.13	0.554	Increase
ENSMUSG00000097391.8	lincRNA	Mirg	miRNA containing gene [Source:MGI Sym	23.7	19.6	23.0	24.3	0.08	0.788	5.65	0.06	0.683	Increase
ENSMUSG00000100755.1	processed_pseudogene	Rps23-ps1	ribosomal protein S23, pseudogene 1 [Sou	6.6	5.0	6.4	6.8	0.09	0.802	5.57	0.00	0.970	
ENSMUSG00000100826.6	processed_transcript	Snhg14	small nucleolar RNA host gene 14 [Source	13.6	10.5	13.4	13.8	0.05	0.820	10.95	0.20	0.153	Increase
ENSMUSG00000097769.7	processed_transcript	Snhg4	small nucleolar RNA host gene 4 [Source:	4.4	3.7	4.4	4.3	-0.02	0.839	1.74	-0.04	0.858	Decrease
ENSMUSG00000087535.2	antisense	Zmiz1os1	Zmiz1 opposite strand 1 [Source:MGI Syn	1.3	0.9	1.2	1.3	0.05	0.842	1.00	0.24	0.406	Increase
ENSMUSG00000092341.2	lincRNA	Malat1	metastasis associated lung adenocarcinor	2862.8	2334.1	2880.3	2845.3	-0.02	0.847	12.61	-0.03	0.801	Decrease
ENSMUSG00000097324.8	lincRNA	Mir143hg	cardiac mesoderm enhancer-associated r	1.8	1.6	1.8	1.8	-0.03	0.859	1.01	0.01	0.982	
ENSMUSG00000055897.13	transcribed_unprocessed_pse	Ppp4r1l-ps	protein phosphatase 4, regulatory subunit	1.2	0.9	1.2	1.3	0.07	0.869	1.71	0.70	**0.012**	Increase
ENSMUSG00000006638.14	processed_transcript	Abhd1	abhydrolase domain containing 1 [Source:	1.1	0.5	1.1	1.1	-0.06	0.871	0.19	-0.15	0.727	Decrease
ENSMUSG00000079179.9	antisense	Rab10os	RAB10, member RAS oncogene family, op	14.0	10.6	13.7	14.2	0.05	0.882	4.35	0.13	0.505	Increase
ENSMUSG00000030424.15	transcribed_unprocessed_pse	Zfp939	zinc finger protein 939 [Source:MGI Symb	3.0	2.2	2.9	3.1	0.06	0.911	2.53	0.05	0.818	Increase
ENSMUSG00000112117.1	lincRNA	Rmst	rhabdomyosarcoma 2 associated transcri	15.5	13.1	15.5	15.5	0.00	0.925	3.31	0.00	0.990	Decrease
ENSMUSG00000080712.3	processed_pseudogene	Hist3h2bb-ps	histone cluster 3, H2bb, pseudogene [Sou	7.8	6.1	7.7	7.9	0.04	0.940	1.03	0.10	0.705	Increase
ENSMUSG00000110834.1	lincRNA	CAAA01180111.2	predicted gene, 39469 [Source:MGI Symb	5.9	4.4	5.9	6.0	0.04	0.941	1.18	-0.04	0.891	
ENSMUSG00000106147.1	snoRNA	Rnu3a	U3A small nuclear RNA [Source:MGI Sym	95.5	48.0	95.7	95.3	-0.01	0.969	2.95	-0.06	0.794	Decrease
ENSMUSG00000093565.1	antisense	Rab26os	RAB26, member RAS oncogene family, op	13.6	9.3	13.5	13.7	0.02	0.985	0.64	0.05	0.904	Increase
ENSMUSG00000082519.1	processed_pseudogene	Vamp7-ps	vesicle-associated membrane protein 7, p	13.3	11.3	13.2	13.3	0.01	0.988	2.69	-0.04	0.807	
